# Literary Note

**Published:** 1911-07

**Authors:** 


### LITERARY NOTE

### Merck's Manual of the Materia Medica

THE fourth edition of Merck's Manual of the Materia Medica has been issued and proves a most valuable book of information. It is a ready reference pocket book for the physician and surgeon. It contains a comprehensive list of chemicals and drugs—not confined to Merck's with their synonyms, solubilities, physiologic effects, therapeutic uses, doses, incompatibles, antidotes, and the like, a table of therapeutic indications, with interspersed paragraphs on bedside diagnosis, and a collection of prescription formulas beginning under the indication "abortion" and ending with "yellow fever;" a classification of medicaments; and miscellany, comprising poisoning and its treatment; and an extensive dose table; a chapter on urinalysis, and various tables. Merck & Co., 45 Park Place, New York, will send a copy on receipt of forwarding charges of ten cents, in stamps, to physicians, or to students enrolled in any college of medicine, in the United States.

